# Role of MicroRNA-124 as a Prognostic Factor in Multiple Neoplasms: A Meta-Analysis

**DOI:** 10.1155/2019/1654780

**Published:** 2019-11-22

**Authors:** Zijian Zhou, Jiancheng Lv, Jingzi Wang, Hao Yu, Hongcheng Lu, Baorui Yuan, Jie Han, Rui Zhou, Xiaolei Zhang, Xiao Yang, Haiwei Yang, Qiang Lu, Pengchao Li

**Affiliations:** ^1^Department of Urology, The First Affiliated Hospital of Nanjing Medical University, Nanjing 210029, China; ^2^Department of Urology, Children's Hospital of Nanjing Medical University, Nanjing 210029, China

## Abstract

**Objective:**

MicroRNA-124 (miR-124) was revealed to be an attractive prognostic tumour biomarker in recent studies. However, the results remain inconclusive. Hence, this meta-analysis was carried out to clarify the precise predictive value of miR-124.

**Materials and Methods:**

Relevant studies were searched in PubMed, EMBASE, Web of Science, and the Cochrane Library up to October 2018. Hazard ratios (HRs) and 95% confidence intervals (95% CIs) were extracted from the selected studies.

**Results:**

A total of 29 articles investigating the correlation between miR-124 expression and prognosis were initially identified. The pooled HR for overall survival (OS) of high miR-124 expression in multiple cancers was 0.55 (95%CI = 0.50–0.61). Disease-free survival (DFS)/progression-free survival (HR = 0.48, 95%CI = 0.38–0.61) revealed a protective role of increased miR-124 expression. Epigenetic hypermethylation of miR-124 mediated the silencing of its expression, which is correlated significantly with unfavourable survival (OS: HR = 2.06, 95%CI = 1.68–2.53; DFS/recurrence-free survival: HR = 2.77, 95%CI = 1.85–4.16).

**Conclusions:**

Taken together, our results suggest that miR-124 plays an antioncogenic role in various tumors, such as lung cancer and colorectal cancer. If methylation of miR-124 could be prevented, progression and metastasis would be improved; thus, miR-124 may be a promising biomarker and novel therapeutic target. Further large-scale studies are needed to confirm this possible effect.

## 1. Introduction

MicroRNAs (miRNAs) are small, single-stranded, and noncoding RNAs. They suppress protein expression through base pairing with the 3′untranslatable region (3′UTR) of target messenger RNA [1, 2]. It is confirmed that the altered expression of miRNAs is involved in diverse biological processes, including cell growth, differentiation, and apoptosis [3]. Emerging articles have demonstrated that miRNAs are upregulated or downregulated in diverse tumours and their expression pattern is tissue-specific. These findings strongly suggest that miRNAs are implicated in human carcinogenesis and cancer progression by functioning as either oncogenes or tumour suppressors.

MiR-124, a brain-enriched miRNA, plays a crucial role in gastrulation, stem cell regulation, and neural development [4, 5]. Growing evidence shows that miR-124 presents low expression in most tumours and high expression in normal tissues. Given its potential tumour-suppressing role, miR-124 may inhibit carcinoma cell proliferation, invasion, and metastasis, resulting in better prognosis. The prognostic value of miR-124 has been noted for helping in the management of patients harbouring cancerous lesions. It benefits from the advancement in molecular biology, especially the analysis of proteins and genes involved in cancer development [6, 7]. However, several studies, such as those on ependymoma, have yielded conflicting results [8]. Thus, the demand to assess the prognostic relevance of the miR-124 in multiple cancers is strong. The present meta-analysis explores the prognostic value of miR-124 expression in diverse cancers.

## 2. Methods

### 2.1. Search Strategies

A systematic literature search was contacted using four electronic databases, including PubMed, EMBASE, Cochrane Library, and Web of Science, until October 2018. The following combined search keywords for literature retrieval were used: (“miR-124” or “hsa-miR-124” or “microRNA-124”) and (“prognoses” or “survival” or “prognostic factors”) and (“tumour” or “cancer” or “neoplasm”). When multiple reports were issued by the same author, only the latest or most intact report was used to avoid overlapping of queues.

### 2.2. Selection Criteria

Criteria for selecting published studies are as follows: (1) English publications, (2) patients who were diagnosed with cancer by pathological methods, (3) association of miR-124 expression with cancer prognosis, and (4) survival outcomes (overall survival (OS), disease-free survival (DFS), recurrence-free survival (RFS), or progression-free survival (PFS)) with hazard ratios (HRs) and 95% confidence intervals (CIs) that could be calculated directly or indirectly.

Criteria for excluding published studies are as follows: (1) non-English papers; (2) no human studies; (3) reviews, cases, reports, meta-analyses, or letters; (4) diagnosis of cancer patients was uncertain; and (5) studies without adequate data to obtain survival outcomes with HRs and 95% CIs.

### 2.3. Data Extraction

Published studies were screened by two reviewers following the established criteria by reading titles, abstracts, and entire texts. If preliminary conclusions were uncertain, the literature was reassessed by all authors. Information extracted from the included studies is as follows: (1) first author's name and publication year; (2) study population of miR-124 high/low expression, nationality, ethnicity, type of disease, and detected sample; (3) detection method, cut-off value, and maximum follow-up time, and (4) HRs associated with upregulated miR-124 expression for OS, RFS, PFS, and DFS, along with their 95% CIs and *p* values. If only Kaplan-Meier curves were available, data were extracted from graphical survival plots to extrapolate HRs with 95% CIs using OriginPro9.0.

### 2.4. Quality Assessment

Two reviewers critically assessed the quality of all studies contained in this meta-analysis. We used the Newcastle-Ottawa Scale to rate the quality of included studies (Table 1). The studies were evaluated in three perspectives: selection, comparability, and outcomes. The range of scores was 0 to 9. Scores of 0–3, 4–5, and 6–9 were, respectively, accepted as low quality, medium quality, and high quality. Only high-quality studies were reflected in this analysis.

### 2.5. Statistical Analysis

The effect of miR-124 expression on the prognosis of diverse cancers was evaluated by pooled HRs with 95% CIs. HR was employed as an indicator of effect size. We selected a fixed-effect model (Mantel-Haenszel method) or a random-effect model (DerSimonian-Laird method) based on the heterogeneity of the pooled studies. Cochran's *Q* test and the Higgins *I*^2^ statistical method were utilized to test heterogeneity; specifically, heterogeneity was confirmed by the former and quantified by the latter. A random-effect model was served if heterogeneity was remarkable (*p* < 0.10 or *I*^2^ > 50%). Otherwise, the fixed-effect model was implemented. Egger's test with a funnel plot was used to assess publication bias. We applied a Galbraith plot to find studies with heterogeneity and then performed the meta-analysis once more after excluding studies with heterogeneity. Moreover, we applied a subgroup analysis to minimise the influence of heterogeneity. Sensitivity analysis was performed by omitting single studies sequentially to evaluate the robustness of pooled results. The two-sided test was applied to calculate all *p* values, and a *p* value less than 0.05 was examined statistically significant. All statistical analyses were performed by using Stata12 (Stata Corp. LP, College Station, Texas, USA) and Microsoft Excel (V.2013, Microsoft Corporation, Redmond, Washington, USA).

## 3. Results

### 3.1. Summary of Enrolled Studies

**Figure 1:**
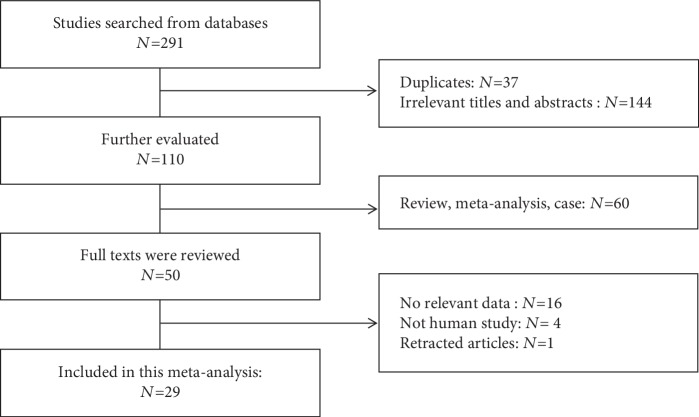
Flow diagram of the study selection process.

A total of 291 studies were collected from our primary literature survey. After removing duplicates, reviews, meta-analysis articles, and articles that were not relevant to the topic or not appraised on patients, 245 records were excluded. In the remaining studies, 16 of them did not contain sufficient survival data (HRs or survival curves) and 1 article had been retracted. Finally, 29 studies were reviewed as eligible for this meta-analysis (Figure 1). The chosen articles were published between 2009 and 2018 and involved different types of cancer: non-small-cell lung cancer (NSCLC, *n* = 7), hepatocellular carcinoma (HCC, *n* = 4), pancreatic cancer (PDAC, *n* = 2), clear cell renal cell carcinoma (ccRCC, *n* = 2), breast cancer (*n* = 2), colorectal cancer (*n* = 2), gastric cancer (*n* = 2), osteosarcoma (*n* = 2), cervical cancer (*n* = 2), ependymoma (*n* = 1), myelodysplastic syndrome (MDS, *n* = 1), glioblastoma (*n* = 1), and acute lymphoblastic leukaemia (ALL, *n* = 1).

A total of 23 studies reported OS, 9 studies focused on DFS, and 3 provided PFS. Five reports explored the relationship between miR-124 methylation and survival. We collected data from the 29 included studies, involving 3,061 participants. The largest sample size was 353, and the smallest was 30. The patients enrolled in 25 studies were Asian; the rest were Caucasian. Patients came from seven countries: China, Japan, Korea, Canada, Israel, Spain, and Germany. Tables 2 and 3 systematically summarize the main features of the 29 enrolled studies.

### 3.2. Analysis of the OS Associated with miR-124 Expression

**Figure 2:**
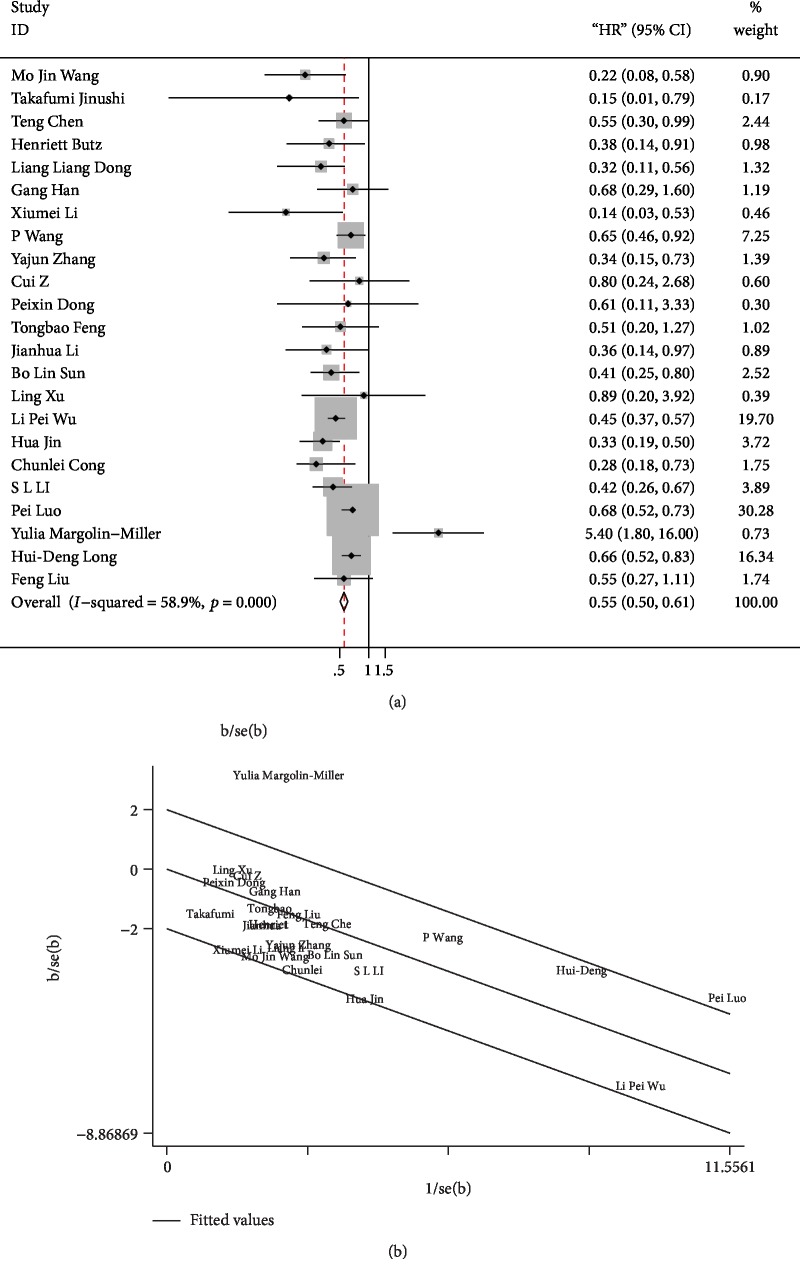
(a) Forest plots of HRs estimated for the correlation between the expression of miR-124 and overall survival (OS). (b) Galbraith plot used to find the cause of heterogeneity in OS.

In total, 23 studies were subjected to OS analysis (Figure 2(a)) with the random-effect model due to its moderate heterogeneity (*p* < 0.001, *I*^2^ = 58.9%). The OS (HR = 0.55, 95%CI = 0.50–0.61) was statistically significant (*p* < 0.001), indicating that high miR-124 expression groups were significantly correlated with enhanced OS and had low death probability. On the Galbraith plot (Figure 2(b)), we found that the articles of Luo et al. and Margolin-Miller et al. were the principle sources of heterogeneity. Heterogeneity substantially decreased when we excluded these two studies (*I*^2^ = 25.7%, *p* = 0.137), and the outcomes remained significant (HR = 0.49, 95%CI = 0.44–0.55). Subgroup analyses were performed to find the main cause of heterogeneity.

### 3.3. Recurrence Associated with miR-124 Expression

Twelve studies included in the DFS/PFS analysis also uncovered a protective role of increased miR-124 expression (HR = 0.48, 95%CI = 0.38–0.61), as determined by the random-effect model (*p* = 0.013, *I*^2^ = 54%). Consistently with the outcome of OS, miR-124 was a favourable prognosis in the analysis. However, heterogeneity was still noted. Similar to the OS, the article of Margolin-Miller et al. was the main cause of this heterogeneity. When we excluded this study, the heterogeneity disappeared (*p* = 0.445, *I*^2^ = 0.00%).

### 3.4. Prognosis with miR-124 Methylation

**Figure 3:**
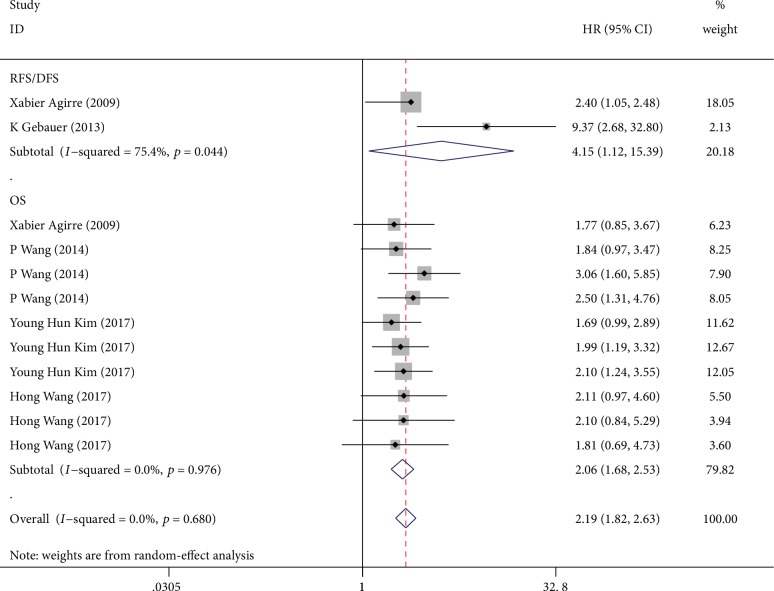
Forest plots of HRs estimated for the correlation between methylation of miR-124 and patient survival.

Figure 3 illustrated a forest plot of the HRs of five studies investigating the relationship between miR-124 methylation and survival (including OS, DFS, and RFS). The overall corrected HR was 2.19 (95%CI = 1.82–2.63), which was statistically significant (*p* < 0.001) in a fixed model. It has been reported that downregulated miRNA expression, resulting from aberrant methylation, could promote the development and progression of several human cancers [9]. Due to the hypermethylation, the expression of miR-124 is downregulated, leading to reduced survival time. We would explain the correlation between miR-124 expression and methylation in the discussion section of this manuscript.

### 3.5. Sensitivity Analyses

**Figure 4:**
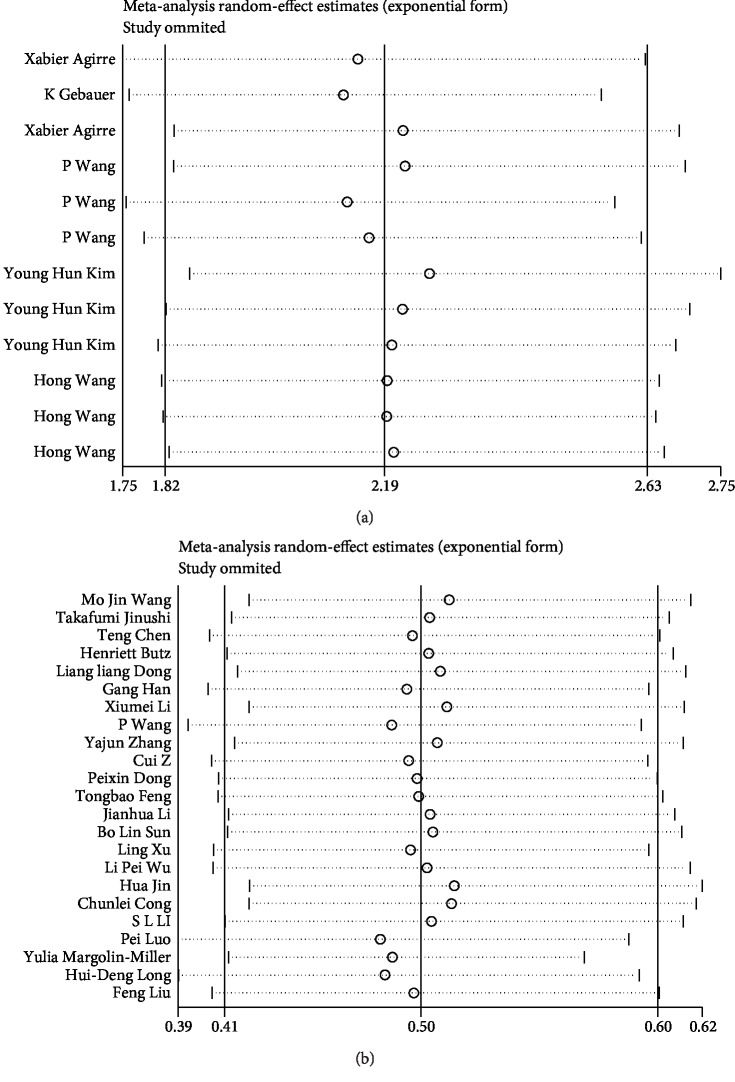
(a) Sensitivity analysis of overall survival. (b) Sensitivity analysis of methylation.

In the OS, DFS/PFS, and methylation studies, our sensitivity analysis results (Figure 4) remained stable. No single study considerably influenced the pooled HRs or 95% CIs. Therefore, the presence of mild heterogeneity may be acceptable.

### 3.6. Publication Bias

**Figure 5:**
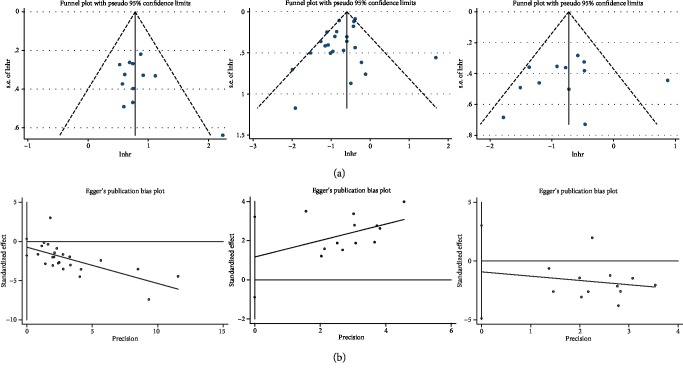
(a) Funnel plot for publication bias. (b) Egger's plot for publication bias.

The funnel plots of publication bias were presented in Figure 5. In the pooled analyses of OS, DFS/PFS, and methylation, Egger's test *p* values were 0.168, 0.162, and 0.234, respectively, as shown by symmetric funnel plots. The funnel plot measures as study size (in this case, standard error) on the vertical axis as a function of effect size on the horizontal axis. Large studies appear towards the top of the graph and tend to cluster near the mean effect size. Smaller studies appear towards the bottom of the graph. Therefore, no evidence of publication bias was noted.

## 4. Discussion

In this meta-analysis, elaborate effort was invested to establish reliable and convincing evidence to evaluate the prognostic impacts of miR-124 expression in patients with carcinomas. Our OS analysis revealed a pooled HR of 0.55, thereby demonstrating that high miR-124 expression was associated with a favourable outcome, and this result was statistically significant (*p* < 0.001). An HR value of 0.48 in the DFS/PFS analysis confirmed our findings again, and it was also statistically significant (*p* < 0.001).

Some of the included studies reported the effects of miR-124 methylation on survival. They found that highly methylated miR-124 genes can be detected in cancer tissues but not in noncancerous tissues. Epigenetic modifications like DNA, RNA, and histone modifications have been proven to be involved in mammalian development, and epigenetic changes were related to different cancers. It was confirmed that hypermethylation mediates the silencing of miR-124 expression [10]. The data showed that miR-124 hypermethylation is correlated significantly with unfavourable survival (OS: HR = 2.06, 95%CI = 1.68–2.53; DFS/RFS: HR = 2.77, 95%CI = 1.85–4.16). This was consistent with the conclusion of the miR-124 expression above. Testing for the presence of miR-124 methylation could help identify patient subgroups at high risk of poor disease outcomes. More intriguingly, several groups delved into the methylation of three members of the miR-124 family, respectively. Three genes of human miR-124 have been identified and located as follows: miR-124a-1 (8p23.1), miR-124a-2 (8q12.3), and miR-124a-3 (20q13.33). In most times, the three genes cooperated and made synergistic effects. In subgroup results of methylation of three genes of miR-124 family, outcomes ([Supplementary-material supplementary-material-1]) were consistent in miR-124a-1 (HR = 1.82, 95%CI = 1.27–2.62), miR-124a-2 (HR = 2.30, 95%CI = 1.59–3.33), and miR-124a-3 (HR = 2.18, 95%CI = 1.50–3.17). As an example, Wang et al. stated that the methylation levels of miR-124a-1, miR-124a-2, and miR-124a-3 were all much higher in PDAC tissues than in normal tissues and they implied that hypermethylation of the miR-124 family was strongly associated with poor prognosis in PDAC patients [9]. Moreover, possibly owing to the different locations of three genes on chromosomes, sometimes they performed their importance. For instance, Gebauer et al. highlighted that miR-124a-3 methylation was an independent prognosticator and associated with disease recurrence of patients with ccRCC, but he did not mention the effect of miR-124a-1 and miR-124a-2 [11]. Conclusively, methylation of three genes of the human miR-124 family all indicated poor prognosis. However, only five articles reported the results of methylation. Additional studies are wanted to confirm the clinical significance of miR-124 methylation in a large number of samples.

Subgroup results in Table 4 supported the above conclusions. Founded on the characteristics of individual studies, we observed statistically significant outcomes in the OS of the NSCLC and HCC subgroups with pooled HRs of 0.43 and 0.56, respectively. Besides, in the subgroup analysis of ethnicity, outcomes were all significant in the Asian (HR = 0.48, 95%CI = 0.41–0.57) and Caucasian (HR = 1.42, 95%CI = 0.11–18.90) groups. Subgroup of “source of HR” showed a significant correlation in the survival curve group (HR = 0.51, 95%CI = 0.39–0.66).

During heterogeneity analysis, OS was proved to be moderately heterogeneous (*p* < 0.001, *I*^2^ = 58.9%). Galbraith plots revealed that the articles of Luo et al. and Margolin-Miller et al. were the foremost sources of heterogeneity. In the article of Luo et al., the HR of OS was obtained from univariate survival analysis, which ignored the combined effects of other factors [12]. Thus, the heterogeneity may exist in his data. Furthermore, high expression of miR-124 may suppress tumours and enhance survival time, but the study of Margolin-Miller et al. did not agree with it. In their article, miR-124-3p, a member of the miR-124 family, was significantly associated with increased risk for the progression of paediatric ependymoma patients. In another two reports, miR-124-3p was highly overexpressed in high-risk paediatric neuroblastoma cases [13, 14]. Therefore, we inferred that miR-124-3p may be an exception in the miR-124 family, and then we collected articles on miR-124 to explore the detailed results. As we found, miR-124-3p directly targeted PDCD6 to inhibit metastasis in breast cancer [15] and cooperated with ROCK1 to reduced procession in Ewing Sarcoma [16]. Luo et al. reported that miR-124-3p suppresses glioma aggressiveness by targeting Fra-2 [17]. In summary, miR-124-3p still plays a suppressive role in several brain tumours (e.g., anaplastic astrocytoma and glioblastoma) [18]. The survival time was reduced only for miR-124-3p in ependymoma and neuroblastoma. These two studies both focused on paediatric tumours while others studied adult tumours. Thus, we extensively studied the connection between miR-124 and paediatric tumours. Lourdusamy et al. compared paediatric spinal and intracranial ependymomas with similar tumours in adults, revealing a relatively low expression of miR-124 in paediatric tumours. In contrast to adult spinal ependymoma (SEPN), downregulated miR-124 in paediatric SEPN was not enriched at the equivalent positions [19]. Therefore, we infer that differences between adults and children may result in heterogeneity. However, to date, only two studies focusing on miR-124 and ependymomas have been published. Hence, further studies are required to expound on this issue.

In the DFS/PFS analysis, Margolin-Miller et al.'s study also resulted in comparable heterogeneity. No single study substantially influenced pooled HRs or 95% CIs. We argued that the mild heterogeneity obtained may result from the categories of the tumours examined and the level of heterogeneity observed is acceptable.

Besides, pri-miR-124 rs531564 polymorphism has been correlated to cancer risk. The functional rs531564 polymorphisms in pri-miR-124 may affect the mature miR-124 amount or function [20]. Previous studies stated that pri-miR-124 rs531564 polymorphism was associated with decreased risk of cancer including cervical cancer [21], esophageal squamous cell carcinoma [22], and CRC [23]. As an example, Gao et al. suggested that pri-miR-124 rs531564 polymorphism contributes to the decreased risk of CRC, poor differentiation, and lymph node metastasis in the Chinese population, possibly by affecting miR-124 expression [23]. However, to our knowledge, no studies clarified the association between pri-miR-124 rs531564 polymorphism and prognostic value clearly. Further work is desired to validate these results in prospective studies and evaluate their prognostic role in clinical practice.

Despite its contributions, this study also presented several inherent limitations. Firstly, heterogeneity was noted during OS analyses. The presence of population heterogeneity may be due to the unique characteristics of the studies, such as the ethnicity of the participants, their nationality, disease type, detected sample, source of HR, and the cut-off value of miR-124 expression. Secondly, the statistical power of the effect of miR-124 methylation was reduced because only five studies related to this topic were enshrined in the present meta-analysis. Thirdly, the lack of global miR-124 expression data makes defining a universal cut-off difficult. Most of the enrolled studies established a median or mean value as the expression cut-off, and these values vary. Therefore, pooled outcomes may be greater or lower than the actual value and cause bias in the results. Fourthly and most recently, “integrated genomics,” which collects information from multiple levels of molecular changes, could increase understanding of the interplay between molecular alterations. The linear combination of several miRNAs, rather than unique miRNAs, should be viewed as a whole to increase predictive power [24]. Finally, only English articles were listed in this meta-analysis, which may cause bias in the results. Subsequent studies are required to address these limitations.

## 5. Conclusion

Our data offer convincing evidence that high expression of miR-124 may be independently associated with a favourable cancer prognosis. Hypermethylation mediated miR-124 downregulation, which was significantly associated with poorer survival of tumour patients. We believe that this meta-analysis is simply the beginning of a sustained exploration of the role of miR-124 in various tumours. More in-depth and larger-scale studies on this topic are needed.

## Figures and Tables

**Table 1:** Newcastle-Ottawa quality assessment scale.

Study	Year	Quality indicators from the Newcastle-Ottawa Scale								Scores
		1	2	3	4	5	6	7	8	
Zheng [25]	2011	★	★	★★	★	★★	★	★	—	9
Wang [26]	2012	★	★	—	★	★★	★	★	★	8
Takafumi [27]	2014	★	★	—	★	★★	★	★	—	7
Chen [28]	2014	★	★	★	★	—	★★	★	★	8
Henriett [29]	2015	★	★	★★	★	★	★	★	—	8
Dong [30]	2015	★	★	—	★	★	—	★	★★	7
Han [31]	2015	★	★	★	★	★	★	—	★	7
Li [32]	2014	★	★	—	★	★★	—	★	—	6
Wang [9]	2014	★	★	—	★★	★	—	—	★	6
Zhang [33]	2015	★	★	★	★	★	—	★	★★	8
Cui [34]	2016	★	—	★	★	★★	—	★	★	7
Dong [35]	2016	★	★	—	★	★	—	★★	★	7
Feng [36]	2016	★	★	★	★	★★	★	★	★	9
Li [37]	2016	★	★	—	★	★	★	★	—	6
Sun [38]	2016	★	★	★	★	—	★	★	★	7
Xu [39]	2016	★	★	★	★	★	★	★	★	8
Wu [40]	2017	★	—	★	★	★	★	★	★	7
Jin [41]	2017	★	★	★	★	—	★★	★	★	8
Cong [42]	2017	★	—	★	★	★	—	★	★	6
Li [43]	2017	★	★	★	★	—	★	★	★	7
Luo [12]	2017	★	★	★★	—	—	★	★	★	7
Yulia [8]	2017	★	★	★	—	★★	★	★	★	8
Long [44]	2018	★	★	—	★	★	★	★	★	7
Liu [43]	2018	★	★	—	★	★	★	—	★	6
Xabier [45]	2009	★	★	★	★	★	★	★	—	7
Gebauer [11]	2013	★	★	★	★	★	★	★	★★	9
Kim [10]	2017	★	★	★	—	★	★	★★	★	8
Wang [9]	2017	★	★	★★	—	★	★	★	★	8

**Table 2:** Essential features and overall survival of the studies contained in this meta-analysis.

Study	Year	High expression	Low expression	OS				DFS/PFS				Nationality	Malignant disease	Source of HR
				HR	LL	UL	*p* value	HR	LL	UL	*p* value			
Zheng	2011	65	66	NM				0.400	0.200	0.800	0.009	China	HCC	Reported
Wang	2012	25	71	0.216	0.081	0.578	0.002	0.221	0.084	0.577	0.002	China	Colorectal	Reported
Takafumi	2014	25	24	0.147	0.008	0.789	0.022	0.624	0.291	1.300	0.209	Japan	Colorectal	SC
Chen	2014	69	68	0.550	0.300	0.990	0.001	0.560	0.320	0.970	0.002	China	Glioma	Reported
Henriett	2015	NM	NM	0.385	0.138	0.912	0.032	0.485	0.169	1.205	0.121	Canada	ccRCC	Reported
Dong	2015	67	66	0.316	0.110	0.559	0.017	NM				China	Breast cancer	SC
Han	2015	53	52	0.680	0.290	1.600	0.005	NM				China	Osteosarcoma	Reported
Li	2014	48	116	0.136	0.034	0.534	0.004	0.168	0.044	0.644	0.009	China	NSCLC	Reported
Wang	2014	32	33	0.652	0.461	0.922	0.015	NM				China	PDAC	SC
Zhang	2015	46	46	0.340	0.150	0.730	<0.05	0.300	0.120	0.730	<0.05	China	NSCLC	SC
Cui	2016	15	15	0.800	0.240	2.680	<0.05	NM				China	NSCLC	SC
Dong	2016	20	20	0.610	0.110	3.330	0.002	0.630	0.150	2.610	0.006	Japan	Cervical cancer	Reported
Feng	2016	24	24	0.506	0.201	1.274	0.015	NM				China	Breast cancer	Reported
Li	2016	57	70	0.362	0.135	0.974	0.044	NM				China	Cervical cancer	Reported
Sun	2016	18	35	0.405	0.247	0.800	0.001	NM				China	PDAC	SC
Xu	2016	20	20	0.890	0.200	3.920	0.017	NM				China	HCC	Reported
Wu	2017	NM	NM	0.452	0.373	0.568	0.002	NM				China	HCC	Reported
Jin	2017	96	99	0.330	0.190	0.500	<0.01	NM				China	NSCLC	Reported
Cong	2017	55	59	0.282	0.177	0.725	0.019	0.255	0.156	0.637	0.012	China	Osteosarcoma	SC
Li	2017	40	48	0.424	0.260	0.670	<0.05	NM				China	Gastric cancer	Reported
Luo	2017	NM	NM	0.680	0.520	0.730	0.023	NM				China	NSCLC	Reported
Yulia	2017	17	50	5.400	1.800	16.000	0.002	2.400	1.000	5.700	0.050	Israel	Ependymoma	Reported
Long	2018	NM	NM	0.659	0.523	0.830	0.036	0.460	0.230	0.950	0.026	China	HCC	SC
Liu	2018	51	70	0.550	0.270	1.110	0.002	0.620	0.330	1.180	0.009	China	Gastric cancer	Reported

NM: not mentioned; SC: survival curve; Colorectal: colorectal cancer.

**Table 3:** Essential features and methylation outcomes of studies used in this meta-analysis.

Study	Year	Methylation	Nonmethylation	OS				RFS/DFS				Nationality	Malignant disease	Type of miR-124	Source of HR
				HR	LL	UL	*p* value	HR	LL	UL	*p* value				
Xabier	2009	208	145	1.770	0.850	3.670	<0.001	2.400	1.050	2.480	<0.001	Spain	ALL	miR-124a	SC
Gebauer	2013	NM	NM	NM				9.3700	2.680	32.800	<0.001	Germany	ccRCC	miR-124-3	Reported
Wang	2014	33	32	1.838	0.973	3.470	0.092	NM				China	PDAC	miR-124-1	SC
Wang	2014	34	31	3.055	1.596	5.850	0.002	NM				China	PDAC	miR-124-2	Reported
Wang	2014	33	32	2.499	1.313	4.757	0.017	NM				China	PDAC	miR-124-3	Reported
Kim	2017	48	109	1.690	0.990	2.890	0.053	NM				Korea	NSCLC	miR-124-1	Reported
Kim	2017	78	79	1.990	1.190	3.320	0.009	NM				Korea	NSCLC	miR-124-2	Reported
Kim	2017	81	76	2.100	1.240	3.550	0.006	NM				Korea	NSCLC	miR-124-3	Reported
Wang	2017	23	33	2.110	0.970	4.600	0.025	NM				China	MDS	miR-124-1	SC
Wang	2017	29	27	2.100	0.840	5.290	0.004	NM				China	MDS	miR-124-2	SC
Wang	2017	35	21	1.810	0.690	4.730	0.010	NM				China	MDS	miR-124-3	SC

**Table 4:** Pooled information for overall survival or disease-free survival/recurrence-free survival stratified by ethnicity and main pathological type for overall and subgroup analyses.

Subgroup	OS					DFS/PFS				
	*N*	HR/95% CI	*p* value	*I* ^2^	PN	*N*	HR/95% CI	*p* value	*I* ^2^	PN
Total	23	0.55 (0.50-0.61)	<0.001	58.9%	2253	12	0.48 (0.38-0.61)	0.013	54.0%	1228
Cancer type										
Lung cancer	5	0.43 (0.25-0.73)	0.006	72.6%	641	2	0.25 (0.12, 0.53)	0.482	0.0%	256
HCC	3	0.56 (0.40-0.78)	0.049	66.6%	307	2	0.43 (0.26, 0.70)	0.782	0.0%	286
Colorectal	2	0.20 (0.08-0.50)	0.763	0.0%	145	2	0.39 (0.14, 1.07)	0.095	64.0%	145
Breast cancer	2	0.39 (0.21-0.71)	0.453	0.0%	181	NM				
Osteosarcoma	2	0.42 (0.18-1.00)	0.119	58.8%	219	1	0.25 (0.13, 0.52)	<0.001	0.0%	114
PDAC	2	0.55 (0.35-0.86)	0.171	46.6%	118	NM				
Cervical cancer	2	0.41 (0.18-0.97)	0.604	0.0%	167	1	0.63 (0.15, 2.63)	<0.001	0.0%	40
Gastric cancer	2	0.46 (0.31-0.68)	0.549	0.0%	209	1	0.62 (0.33, 1.17)	<0.001	0.0%	121
ccRCC	1	0.38 (0.15-0.99)	<0.001	0.0%	62	1	0.49 (0.18, 1.30)	<0.001	0.0%	62
Ependymoma	1	5.40 (1.81-16.10)	<0.001	0.0%	67	1	2.40 (1.01-5.73)	<0.001	0.0%	67
Glioma	1	0.55 (0.30-1.00)	<0.001	0.0%	137	1	0.56 (0.32, 0.97)	<0.001	0.0%	137
Ethnicity										
Asian	21	0.48 (0.41-0.57)	0.015	44.7%	2124	10	0.42 (0.33, 0.55)	0.360	9.0%	1099
Caucasian	2	1.42 (0.11-18.90)	<0.001	92.2%	129	2	1.10 (0.23, 5.26)	0.017	82.5%	129
Source of HR										
Reported	16	0.48 (0.37-0.61)	<0.001	71.9%	1600	7	0.45 (0.24, 0.83)	0.001	72.4%	683
Survival curve	7	0.51 (0.39-0.66)	0.861	0.0%	653	5	0.51 (0.37, 0.71)	0.743	0.0%	545

PN: patient numbers; Colorectal: colorectal cancer.

## Supplementary Materials

Supplementary Materials Forest plots of HRs estimated for the correlation between methylation of three genes of the miR-124 family and OS.
