# Supplementary figures and images for: Role of MicroRNA-124 as a Prognostic Factor in Multiple Neoplasms: A Meta-Analysis
Source: Dis Markers. 2019 Nov 22;2019:1654780. doi: 10.1155/2019/1654780 (PMC6893269; doi:10.1155/2019/1654780)

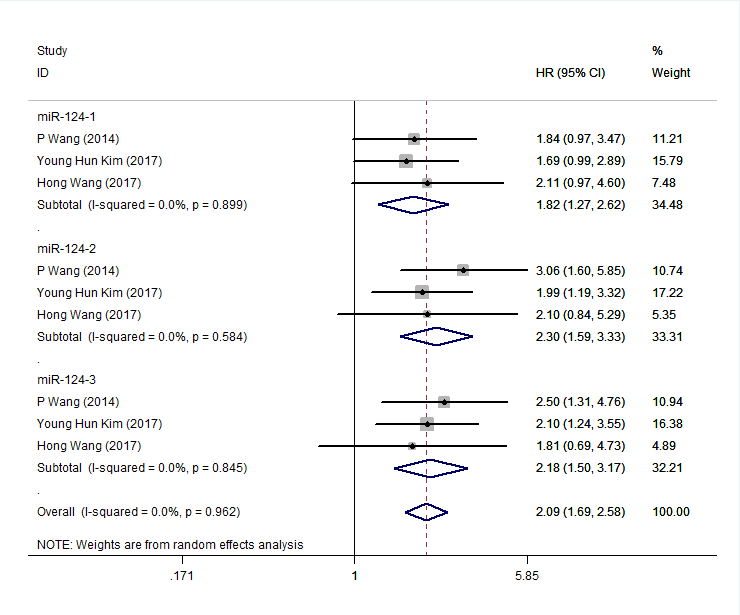

Supplement: Supplementary Materials — Forest plots of HRs estimated for the correlation between methylation of three genes of the miR-124 family and OS. [file 1654780.f1.tif]
